# Wireless Communication Technologies for Safe Cooperative Cyber Physical Systems

**DOI:** 10.3390/s18114075

**Published:** 2018-11-21

**Authors:** Ali Balador, Anis Kouba, Dajana Cassioli, Fotis Foukalas, Ricardo Severino, Daria Stepanova, Giovanni Agosta, Jing Xie, Luigi Pomante, Maurizio Mongelli, Pierluigi Pierini, Stig Petersen, Timo Sukuvaara

**Affiliations:** 1 Innovation, Design and Technology (IDT), Mälardalen University, 72123 Västerås, Sweden; 2 RISE SICS Västerås, Stora Gatan 36, 722 12 Västerås, Sweden; 3 CISTER Research Centre, ISEP, Polytechnic Institute of Porto, 4249-015 Porto, Portugal; aka@isep.ipp.pt (A.K.); rarss@isep.ipp.pt (R.S.); 4 The Department of Information Engineering, University of L’Aquila, 67100 L’Aquila, Italy; dajana.cassioli@univaq.it (D.C.); luigi.pomante@univaq.it (L.P.); 5 DTU Compute, Technical University of Denmark, 2800 Kongens Lyngby, Denmark; fotisf@dtu.dk; 6 Space and Earth Observation Centre, Finnish Meteorological Institute, 99600 Sodankylä, Finland; daria.stepanova@fmi.fi (D.S.); timo.sukuvaara@fmi.fi (T.S.); 7 Dipartimento di Elettronica, Informazione e Bioingegneria, Politecnico di Milano, Via G. Ponzio 32, I-20133 Milano, Italy; agosta@acm.org; 8 Group Technology & Research, DNV GL, Veritasveien 1, 1363 Høvik, Norway; jing.xie@dnvgl.com; 9 CNR-IEIIT, via De Marini 6, 16149 Genova, Italy; maurizio.mongelli@ieiit.cnr.it; 10 Innovation and Technological Services (ITS), Intecs S.p.A., 56121 Pisa, Italy; pierluigi.pierini@intecs.it; 11 SINTEF ICT, 7465 Trondheim, Norway; Stig.Petersen@sintef.no

**Keywords:** cooperative cyber-physical systems, wireless communication, safety, reliability, 5G

## Abstract

Cooperative Cyber-Physical Systems (Co-CPSs) can be enabled using wireless communication technologies, which in principle should address reliability and safety challenges. Safety for Co-CPS enabled by wireless communication technologies is a crucial aspect and requires new dedicated design approaches. In this paper, we provide an overview of five Co-CPS use cases, as introduced in our SafeCOP EU project, and analyze their safety design requirements. Next, we provide a comprehensive analysis of the main existing wireless communication technologies giving details about the protocols developed within particular standardization bodies. We also investigate to what extent they address the non-functional requirements in terms of safety, security and real time, in the different application domains of each use case. Finally, we discuss general recommendations about the use of different wireless communication technologies showing their potentials in the selected real-world use cases. The discussion is provided under consideration in the 5G standardization process within 3GPP, whose current efforts are inline to current gaps in wireless communications protocols for Co-CPSs including many future use cases.

## 1. Introduction

Modern embedded systems, coupled with the advancements of digital communication technologies, have been enabling a new generation of systems, tightly interacting with the physical environment via sensing and actuating actions: Cyber Physical Systems (CPS) [1]. These systems, characterized by an unprecedented level of pervasiveness and ubiquity, have been increasingly relying on wireless communication technologies to provide seamless services for the Internet of Things (IoT) [2] and Industry 4.0 [3], via flexible cooperation. As these Cooperative CPS (Co-CPS) starts approaching safety-critical application domains (e.g., automated vehicles platooning in the automotive and maritime domains, process control in hazardous industries, etc.), safety shows-up as a crucial topic that must be carefully analyzed because failures and errors might lead to hazardous situations, e.g., death, injuries or environmental damages. All these systems are required to perform specific safety functions to ensure that the risk of system’s failure is maintained at an acceptable level. What makes the safety of these systems even more challenging is the fact that they heavily rely on wireless communication to exchange safety-critical information. For example, in automotive applications like vehicular platooning [4,5,6], the IEEE 802.11p standard is used as a communication protocol among vehicles, in a closed-loop control system where exchanged messages contribute to maintain the inter-vehicle safety distance. Message losses or delays may lead to serious crashes among the vehicles in the platoon with dramatic consequences. In this case, real-time and reliability are two important aspects for ensuring the safety of operation of the platoon. Furthermore, security is also very relevant as any possible attack on the platoon, such as for example false data injection, spoofing or jamming, would lead to disastrous consequences as well.

For several years, topics such as safety in Co-CPS have been mostly ignored to the point that, currently, the absence of a de-facto standard on safe and secure Co-CPS is becoming an impediment to their adoption. Security, on the other hand, has been investigated to some extent in several protocols like IEEE 802.11p, IEEE 802.15.4 [7,8] and its variants [9]. However, there are still several challenges in what concerns the impact analysis of several attacks upon cooperating functions, or the integration of security mechanisms with remaining Quality of Service (QoS) properties for safety assurance.

In this line, the ECSEL SafeCOP EU research project addresses these issues regarding the design of wireless safe and secure Co-CPS [10]. In fact, the SafeCOP project deals with safe cooperation of complex CPS that relies on wireless communications. The main objective is to provide a *safety assurance methodology* for these systems with emphasis on applications in the healthcare, maritime, automotive domains.

In this paper, we provide an overview on the safety requirements, challenges and solutions of Co-CPSs relying on wireless communications. We review the state-of-the-art of standard protocols used in Co-CPSs and assess their compliance to the requirements of safety, security and real-time for Co-CPSs. The main result provided by this survey is a collection of general recommendations for decision-making about the use of wireless technologies in Co-CPSs, which are illustrated through the application in relevant real-world use cases. In addition, given that currently 5G constitutes one of the most important areas of research in Europe towards enabling the interoperability and cooperation of heterogeneous radio technologies for the provision of innovative powerful services, we discuss how the current efforts in 5G standardization contribute to addressing the Co-CPSs challenges.

Several reviews of CPS are already presented in the literature, since 2011 [11], with focus on specific issues, such as: system design [12], open source [13], medical applications [14], testing [15], industry 4.0 [16], interoperability [17] and monitoring with formal verification [18]. Many others address security and inherent countermeasures, e.g., [19,20]. Some others focus on energy topics (smart grids and building, wind plants) [21,22,23]. Only two others are directly comparable to the work presented here as they concern safety [24,25]. Our focus is new as we address how to overcome impairments on safety due to the wireless channel with reference to real testbeds.

The rest of the paper is organized as follows: Section 2 provides the state of the art of Co-CPS as technical background. Section 2.5 outlines different Co-CPS use cases that are addressed in the SafeCOP project. Section 3 provides a survey on wireless communication technologies used in Co-CPSs pointing also out their safety issues, predicting how these could be overcome. Section 4 provides a survey about the safety and security protocols for the considered Co-CPS use cases. Finally, Section 5 explores the current 5G standardization efforts and shows to what extent new 5G standard proposals solve the Co-CPS challenges in each SafeCOP’s use case, drawing out from the previous chapters. Section 6 concludes the paper.

## 2. Technical Background

In the SafeCOP project, we are interested in the class of “safety-critical Co-CPSs” [26,27] where any failure may provide damages of different types and severity (financial, environmental, health, human life, etc.). Thus, the concept of safety is related to the capability of controlling and mitigating hazardous situations. Such systems highlight significant challenges that are not adequately addressed by existing practices, related to their rapid design, development and integration, under (non-functional) requirements of safety, security and dependability. Furthermore, mechanisms and methods for efficiently upgrading and re-certifying systems are needed.

Dependability, as a general term, takes care of several aspects like performance, fault tolerance, adaptiveness to unpredictable environment evolution, CPS platea variability and reconfiguration, issues affecting the communications reliability, etc. There are many subtle interactions and interdependencies among safety, security and dependability (also mentioned as Quality of Service—QoS). Often, security is conflicting with safety and QoS, thus requiring to evaluate a quantifiable trade-off between the three. Security is a composite of several attributes including availability, confidentiality and integrity [28]. The introduction of security requirements into systems tends to modify the priorities of some other non-functional requirements. In addition, resource constraints may make it infeasible to guarantee absolute security in all circumstances.

Developing a safety critical system, thus, typically requires making design decisions that trade-off safety concerns, functionality, cost, and other considerations. Achieving adequately safe cooperative cyber-physical systems requires arriving at realising, and assuring a safe design even though participants in the design process are competitors reluctant to share all of their concerns or intricacies of designs with each other. Moreover, due to the cooperative and openness nature, many circumstances which have to be covered by the pre-release safety assurance are difficult to anticipate at design time in the case of Co-CPS.

### 2.1. Co-CPSs Safety Definitions

A wide variety of technical approaches and methods have been used or proposed to analyse system safety, hazards and risk over several decades. The concept of risk management is addressed by ISO 31000 [29] standard that provides a generic framework for assessing and managing risk across various industries. The aim is to obtain an understanding of the risk to inform decisions regarding whether risk is tolerable with respect to some criteria, to differentiate risk associated with different options/decisions, and to determine if (and which) risk treatment options should be implemented to control or modify risk. Barrier management is a safety philosophy widely used in the oil and gas industry [30]. The idea is to control risk by putting measures in place to prevent undesirable incidents from occurring and limit their effects if they occur. Barriers intended to reduce the likelihood of undesirable incidents are called preventive barriers, whereas barriers implemented to avoid escalation and reduce effects of incidents are called mitigating barriers. Systems-Theoretic Accident Model and Processes (STAMP) is a recent accident model, first introduced by [31], based on systems theory focusing on enforcing behavioural safety constraints rather than preventing failures. STAMP is able to assess complex sociotechnical systems by thinking of safety as a control problem rather than a reliability one. Failure Modes, Effects, and Criticality Analysis (FMECA) (a variant of FMEA adding the assessment of criticality) originated from the U.S. Military and was first described in a Military procedure MIL-P-1629A [32] and later used by NASA in the Apollo program. An FMECA involves reviewing components, sub-systems and assemblies to identify failure modes, causes and effects. The approach is described in [33]. Other significant approaches are the Fault Tree Analysis (FTA) and the Event Tree Analysis (ETA).

Looking at the industrial context, several standards are applied. The IEC61508 [34] is an international standard of rules applied in industry. It defines functional safety as part of the overall safety relating to the EUC (Equipment Under Control) and the EUC control system which depends on the correct functioning of the E/E/PE safety-related systems, other technology safety-related systems and external risk reduction facilities. Central to the standard are the concepts of risk and safety functions. The risk is a function of the frequency (or likelihood) of the hazardous event and the event consequence severity. The risk is reduced to a tolerable level by applying safety functions that may consist of E/E/PES and/or other technologies. IEC 61508 defines safety integrity level (SIL) as a discrete level (one out of possible four), corresponding to a range of safety integrity values, where SIL 4 is the highest level of safety integrity and SIL 1 is the lowest. The standard has its origins in the process control industry. It covers the complete safety life cycle, and may need interpretation to develop sector-specific standards. In fact, the standard lies at the root of a number of specific domains e.g., IEC26262 for automotive, EN50128 for railway applications, IEC60601 for medical devices, etc.

### 2.2. Safety Approach in SafeCOP

The development of Co-CPS poses challenges on safety issues that are not adequately addressed by existing practices and standards exposed above. One of the primary objective of SafeCOP is to propose an approach to the safety assurance of Co-CPS which will facilitate their certification and market release.

The system’s safety behavior is typically modeled through a set of “safety cases”. A safety-case is a well-documented body of evidence, in the form of a clear argument, assuring that the system is acceptably safe. Building the safety case requires ensuring not only that identified failures have been addressed, but also that any unwanted interactions between the system parts as well as the environment have been managed. This is usually accomplished by gathering the risk assessment’s results, i.e., the safety evidence during system development.

To obtain this result, we propose a combination of:

- a safety-assurance framework for Co-CPS,
- a reference “Runtime Manager” is able to detect at runtime abnormal behaviour, triggering, if needed, a safe degraded mode.

SafeCOP applies the Safety Assurance approach to manage functional safety activities during the life-cycle of the machine, i.e., the “assumption/guarantee contracts” that facilitates compositional verification and allows for independent development of components. As the contracts capture safety-relevant behaviors, they are used during system development for generating system-specific safety cases. It is necessary to assure that the system relates to the runtime assurance claim of whether the system is still sufficiently safe (whether the contracts are violated) in the current environment or not. A continuous Runtime Manager checks for contract violations. Since contracts are the specifications of the system’s behavior, contract violations are seen as the system failures. Contracts must be always satisfied in any environment condition, thus their violation during runtime indicates that a failure occurred in the environment, i.e., the behavior guaranteed at design-time has been broken. On the other hand, if the contract assumptions are not violated, then the runtime manager should check if the system offers the promised guaranteed behavior. If the guaranteed behavior is not provided, then an internal failure exists.

### 2.3. Security vs. Wireless Communication

Beyond the safety, the overall system dependability relies on security: there are many subtle interactions and interdependencies among safety, QoS and security as introduced in previous sections. Often, security is conflicting with safety and performances, thus requiring to evaluate a quantifiable trade-off between the three. Security is a composite of several attributes including availability, confidentiality and integrity [28]. The introduction of security requirements into systems tends to modify the priorities of some other non-functional requirements. In addition, resource constraints may make it infeasible to guarantee absolute security in all circumstances. One of the key issues that allows the trade-off evaluation is the definition of a metric [35,36]. Metrics are also suitable for security assessment of services, applications, as well as users and communication channels. Several strategies have been proposed in the area of communication channels security to secure protocols and messaging schema. The most widely used mechanism over TCP/IP networks is currently the Secure Socket Layer (SSL), a cryptographic protocol that provides data authentication, encryption and integrity. A SSL connection is established, between two pair nodes, exchanging identification parameters in the form of digital certificates. Defense mechanisms against possible threats, either malicious or due to environment, are also defined. In particular, wireless communications are subject to physical layer attacks, like, e.g., the well-known family of wireless jamming attacks (a noise burst that may result in a Denial of Service (DoS) attack on a wireless channel), or, the most common eavesdropping attacks. These require specific measures known as *physical layer security mechanisms* aimed at increasing the robustness and secrecy capacity of the wireless channel [37]. The main issues are related to the enhanced flexibility and scalability of the networks, especially in the case of Co-CPSs, where different systems could participate to a cooperative group with different roles and the group dimension can vary over time, like in vehicular use cases, where a platoon is a high dynamic set of cars continuously entering and exiting the platoon itself. Vision and details on these items are available in, e.g., [38,39,40]. While the security approaches proposed in the literature mainly focus on the trustiness of the information flowing through the network, enforcing the network access and utilization (e.g., user authentication, message integrity, etc.), the evolutionary scenarios for Co-CPSs require that the trustiness of services and users must also be guaranteed, extending the authentication mechanisms.

### 2.4. Security Approach in SafeCOP

SafeCOP aims to extend the current wireless protocols for both safe and secure cooperation. SafeCOP propose an application-level “safety layer” on top of existing protocols to ensure safe and secure cooperation such that Co-CPS can be certified.

The wireless communication channels considered in SafeCOP and addressed by Use Cases span from 802.11p (for automotive domain), 802.11 for generic communication support, 802.15x for short range communication, as well as mobile LTE. In general, such technologies have been designed to meet communication requirements and significant progress has been done to secure the channels, but they cannot satisfy the safety requirements imposed by the selected use cases. Therefore, SafeCOP is working to enhance current wireless communication protocols to ensure that safety requirements are preserved, together with security, in the highly dynamic scenarios envisaged for Co-CPSs, where traditional safety assurance methods may not be sufficient. Details on communication technologies adopted by each use case and the protocol enhancements proposed within SafeCOP project are presented in Section 3 and Section 4.

However, attention is taken to the technology evolution of mobile networks since the future 5G technologies will address most of the challenging network issues, e.g., providing higher bandwidth, very low latency, specific priority schemes, device-to-device (D2D) communications, as well as more mobile-specific capabilities. Network virtualization increases the flexibility of 5G networks, and improves their adaptability to the specific communication requirements of Co-CPSs. While in the multi-provider environments of current mobile networks communication services are conveyed by different providers, the 5G ecosystem allows a further degree of flexibility through virtualization: application functions and services fall in the paradigm of “Everything as a Service” of cloud computing. Moreover, the storage capability of the cloud allows to collect a wide range of (sensors) data and to support the application logic with data analytics. A deeper discussion on 5G is available in Section 5.

### 2.5. SafeCOP Use Cases

**Figure 1:**
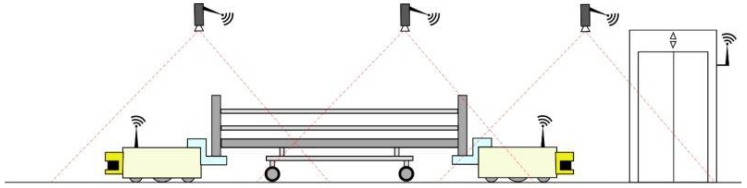
Layout of the use case “Autonomous Hospital Beds”.

**Figure 2:**
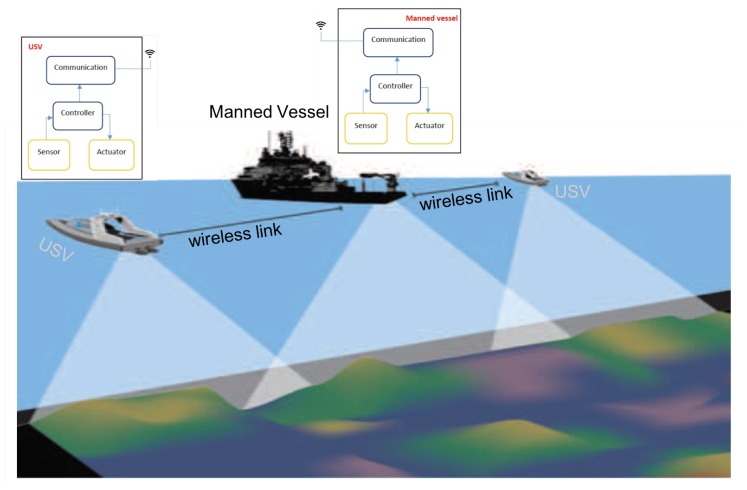
During the bathymetry measurements operation, the boats and the unmanned surface vehicle (USV) communicate wirelessly for coordination.

**Figure 3:**
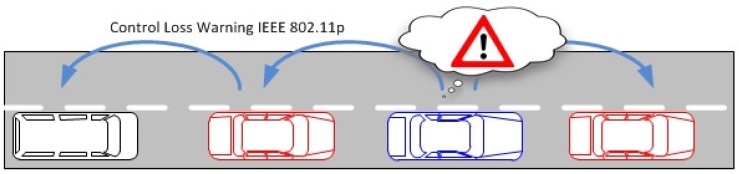
Vehicle control loss warning.

**Figure 4:**
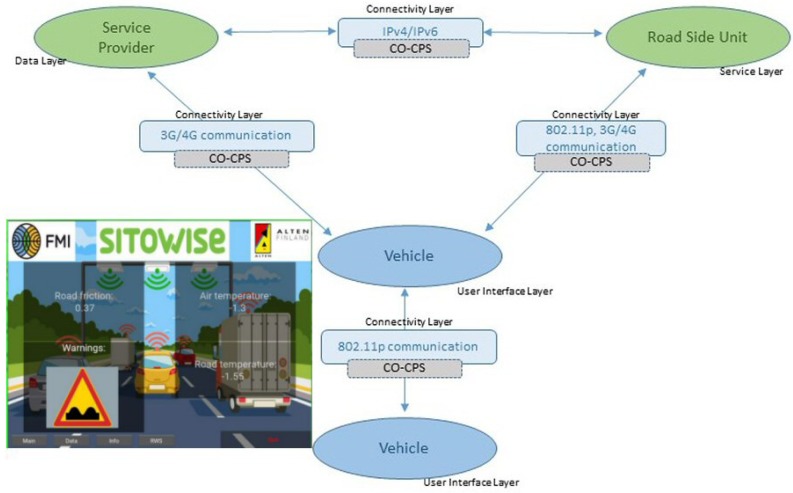
Operational structure of the use case “Vehicles and Roadside Unit (RSU) interaction” with communication to the cloud using 3G/4G, and IEEE 802.11p and 3G/4G for communication between vehicles and roadside units.

**Figure 5:**
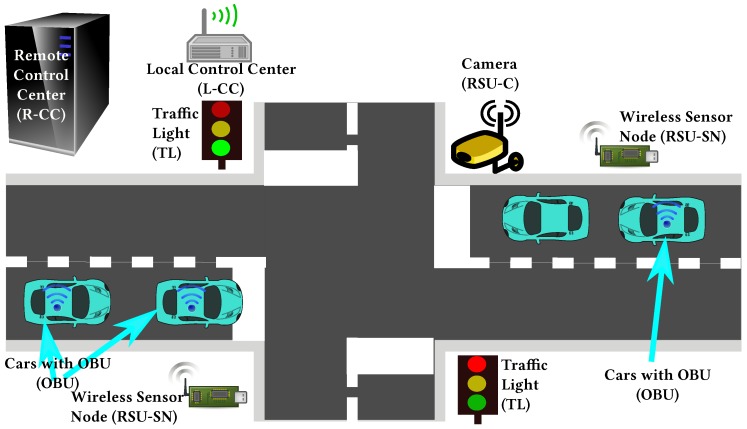
Typical scenario for a V2I cooperation system for traffic management.

**Figure 6:**
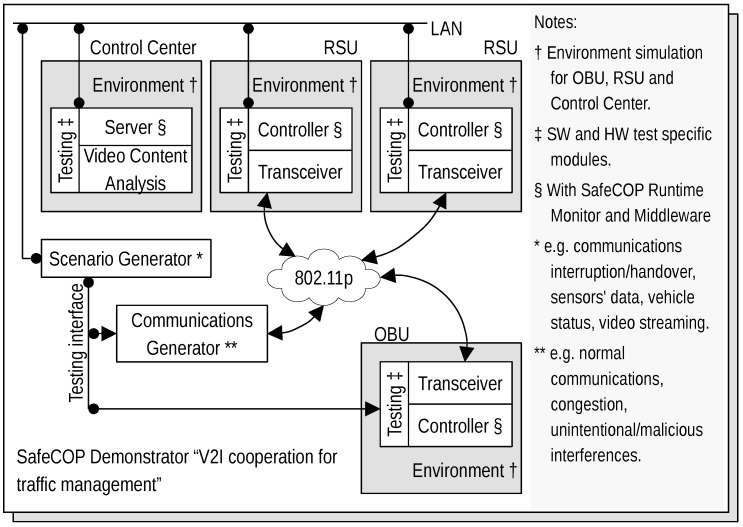
System architecture for the traffic management application through V2I cooperation.

The SafeCOP project aims to cover safety-crytical Co-CPS of a wide range of industrial domains. To reach this objective it is driven by five representative use cases that span from hospital applications with low-speed movements, vehicular applications with high speed movements, to maritime applications. In what follows, we briefly introduce the use cases addressed in the SafeCOP project.

(A) **Hospital Application: Autonomous Hospital Beds**
  To reduce the risk of contamination and spread of disease, hospital beds are thoroughly cleaned before being used by a new patient. In most hospitals, the cleaning is performed manually on site in each patient room, even in hospitals where they have a centralized bed-washing facility (CBWF). This is because moving the bed to and from the CBWF takes about the same amount of time as cleaning the bed in the ward. Both manually cleaning a bed and transporting beds to the CBWF are tasks that require hard physical labor and non-ergonomic motions and positions. To avoid unnecessary strain on hospital workers, and to free up a large amount of their time, this use case proposes an automated solution using two small mobile robots, designated MiR00. After the discharge of a patient, the MiR100s will autonomously transport the bed from the ward to the CBWF for washing, and then bring a clean bed from the CBWF back to the ward. A MiR100 will be attached to each end of the bed, and coordinate and synchronize their movement through the use of safe communication. As the MiR100s are small, their vision is quite limited. To assist in navigation and obstacle detection, a network of cameras will be installed in the hospital hallways. These cameras will act as remote eyes, allowing the robots to maneuver in restricted spaces and keep out of peoples’ way.
  Figure 1 provides the layout of the hospital beds testbeds, where two individual mobile robots are moving in the corridor covered by wireless access points.
(B) **Maritime Application: Autonomous Boat Platoons**
  Since international shipping is responsible for approximately 90% of world trade transportations, the safety of vessels is critical to the global economy. Human errors account for approximately 75% of the almost 15,000 marine liability insurance claims analyzed over five years, which correspond to over $1.6 bn [41]. Autonomous/semi-autonomous ships could improve maritime safety but revolutionize the movement of ships [42]. International Maritime Organization (IMO, London, UK) has received a proposal supported by a number of countries to include autonomous ships on its agenda. The IMO Maritime Safety Committee will establish a new international legal framework for the safe operation of autonomous vessels. It is evident that safety considerations are crucial in this respect. The main barrier to the development of autonomous shipping is represented by the concerns related to the risk of collision between manned and unmanned vessels. Moreover, as the number of cyber threats are increasing, a great concern is raised on specific cyber-attacks targeting the control of autonomous vessels [43]. To reduce inherent risk, cybersecurity should be taken at a high priority when developing autonomous ships.
  Among various explorations of autonomous ships, bathymetry (Bathymetry is the study of underwater depth of lake or ocean floors. Bathymetric charts are typically produced to support geophysical exploration and environmental monitoring) is a very attractive application [44]. Bathymetry is usually performed by sailing a boat with a multi-beam sonar in a rather repetitive lawn-mover pattern. The data acquisition should ideally be going on 24/7, but when using manned survey boats this possibility may be limited due to crew Health and Safety Executive (HSE) regulations. This is an ideal task for an unmanned surface vehicle (USV) that sails these repetitive patterns 24/7. USV application to bathymetry will result in a twofold gain: saving costs and reducing HSE risk for survey personnel [45]. This gain increases for bathymetry measurements in extreme conditions like the Arctic Ocean, where USV may replace a fully-crewed ship and shows better performance in adverse environments and inclement weather.
  At the current stage, the USV has to be remotely controlled by a human operator who is located on another vessel. Such cooperation between the USV and the manned vessel can dramatically increase navigation safety while heavily relying on wireless communications between them, as illustrated in Figure 2. The USV and manned vessel have to periodically exchange critical information, such as vessel speed, course and position, to maintain a certain formation. The USV receives instructions and commands from the manned vessel to maneuver or stop. When safety-critical events occur (e.g., potential collisions), the manned vessel sends safety control commands to the USV, which has to respond within a certain time to avoid collisions. Therefore, packets carrying critical information and safety control commands are subject to *very low latency requirements*.
(C) **Vehicular Applications**
  Safety, comfort and efficiency of both roads and vehicles have improved considerably over the last decade. However, our transportation system still suffers from many problems. The fast growth of urban areas causes an increasing trend of vehicular traffic and road accidents, resulting in serious socioeconomic problems. According to the latest report from the United States (U.S.) National Highway Traffic Safety Administration (NHTSA, Washington, DC, USA), the annual casualties of motor vehicle crashes was a total of 32,999 fatalities and 3.9 million injuries on the roadways of the U.S., which is equal to the annual economic loss to $836 billion [46]. Moreover, in 2014, highway users in the U.S. spend extra unnecessary 6.9 billion hours in traffic jams and consume an additional 3.1 billion gallons of fuel, adding up to an annual economic loss of $160 billion [47].
  To address these problems, there have been worldwide efforts by automotive companies, universities, and governments to provide applications, services, and technologies that connect a vehicle to its surroundings. Examples of such applications and services may include adaptive cruise control, automate braking, remote vehicle diagnostics, hazards, and blind spot warnings. Typically, a connected vehicle (CV) includes interactive advanced driver-assistance systems (ADAS) and cooperative intelligent transport systems (C-ITS), where vehicle awareness concerning its current traffic context is aided by information exchange with surrounding vehicles through vehicle-to-vehicle (V2V) communication, close roadside units through vehicle-to-infrastructure (V2I) communication or people through vehicle-to-pedestrian (V2P) communication, collectively referred as V2X. The use of V2X communications can expand the horizon of on-board sensing systems, thereby eliminating 80% of the current road accidents and providing a smarter and safer ground transportation system [48]. These technologies are anticipated to offer significant benefits, including: reduced driver stress and possibility for passengers to rest and work while traveling; reduced driver costs of paid drivers for taxis and commercial transport; mobility for non-drivers including disabled people, therefore reducing the need for motorists to chauffeur non-drivers, and to subsidize public transport; increased road safety and therefore crash costs and insurance premiums; reduce high-risk driving, such as when impaired e.g., by alcohol consumption; efficient parking, increasing motorist convenience and reducing total parking costs; increase fuel efficiency and reduce pollution emissions. SafeCOP defined three use cases related to the vehicular applications, as described in the following.
  - Vehicle Control Loss Warning
    The goal of this use case is to demonstrate how we can apply and extend wireless technologies to support automotive cooperative V2x-based systems such as auto-braking in vehicle platooning. Besides inter-vehicle networking, this use case is also exploring intra-vehicle communication. Therefore, we consider a scenario where a platoon of vehicles is traveling along a motorway, and Control Loss Warning (CLW) system should be able to detect any safety relevant occurrence that may compromise the vehicle’s platooning ability, such as a braking system failure. Upon detection, the system should send a CLW alert to the other elements involved in the process, e.g., other cars in the platoon.
    The operation in this use case is illustrated in Figure 3. In case of control loss of any vehicle (the blue vehicle in the figure), CLW alert is delivered from car to car forward and backward using the V2V communication infrastructure, and eventually each vehicle gains knowledge about the CLW and can react in a pre-defined manner, by entering in a safe mode. In addition, a wireless network of in-vehicle sensors and actuators is exchanging data with the on-board unit to inform about the status of different automotive systems.
  - Vehicles and Roadside Unit (RSU) interaction
    This use case has been built upon the data exchange between the roadside road weather station and a passing vehicle. Road weather stations (RWS) are typically installed to fixed locations beside the road, collecting different measurement parameters related to weather and traffic, and delivering this data to a single data collection point, typically being the road administrator. Within its operative RWS and vehicular measurement entity, FMI demonstrates this operational environment [49]. During the RWS pass, the vehicle receives up-to-date local road weather information. As an exchange, vehicle can also deliver its own observational information back to RWS, to be used as local supporting data in meteorological services. In this vehicle-roadside unit interaction, we must ensure that the delivered data is not altered or violated by a third party or some communication malfunctioning.
    The primary scenario in this use case is data exchange between vehicle and RWS, when and where the vehicle is passing the RWS. The basic scenario is introduced in [50]. This scenario is extended to cover also data exchange between two vehicles (scenario 2). In this case, both vehicles share the data received from the latest RWS, and as a result both vehicles will obtain up-to-date road weather data from the area ahead. The communication in this scenario is naturally local area communication. In the final extension (scenario 3), we employ an IoT cloud as communication entity, and instead of direct data exchanges between vehicles and RWSs, the IoT cloud shares the location-based relevant data with vehicles and RWSs. The architecture of these resulting three scenarios are presented in Figure 4. A vehicle and a roadside unit are participating in the first scenario (vehicle-to-infrastructure), two vehicles in the second scenario (vehicle-to-vehicle) and, finally, a service provider, a vehicle and a roadside unit to the final (third) IoT-cloud scenario.
  - V2I Cooperation for Traffic Management
    This use case aims at providing an innovative platform that integrates into the V2I network both traffic management (TM) and Video Content Analysis (VCA) functionalities, as shown in Figure 5. VCA consists in the acquisition from video cameras (referred to as RSU-C in Figure 5) and subsequent elaboration through appropriate algorithms. Active road safety (ARS) programmes will strongly benefit from this integration, which enables, via VCA, the early-detection of possible dangerous road events/situations (e.g., vehicles slowing down, vehicles queue, motionless objects) and, via TM applications, the fast drivers’ alert of such traffic anomalies [51,52].
    The probability of traffic accidents will decrease by providing assistance to drivers exploiting both ARS (e.g., collision avoidance systems) and other management applications, like Adaptive Traffic Light Systems (A-TLSs) and dedicated wireless sensor networks (WSNs). A-TLSs change the traffic lights signaling plan (the duration of red, yellow and green phases) according to a set of control parameters, e.g., the time and the day. A-TLS improvements enabled by VCA allow the optimization of the signaling plan according to the changing traffic conditions, usually by extending the green phase when vehicles are closely spaced.
    Figure 6 shows the architecture of the envisaged system. It integrates several SafeCOP framework components, including runtime mechanisms for safety assurance and distributed safety-critical cooperation techniques (based on extensions to IEEE 802.11p), into a Traffic Management Application, which runs in a distributed way. This system will represent one of the SafeCOP demonstrators. It is composed of on-board (OBU) and road side (RSU) units, and a server-based Control Center. Communications between the parts of the demonstrator system are performed through radio frequency front-ends which transmit and receive on-the-air, or through attenuators and noise generators for testing purposes. The OBU integrates radio communication and inertial sensors, allowing additional information on vehicle behavior to be received by the RSU. The RSU acquires video from the camera and performs the necessary initial elaboration to reduce communication times, aggregates information from vehicles in its operating range, and transmits the information over a wired connection to the remote Control Center.

## 3. Communication Technologies for SafeCOP Co-CPSs

Communication technologies vary for different Co-CPS use cases. For different use cases, we can use different protocols, open and proprietary, and technologies. Here, we give a brief overview of the most common communication technologies and protocols in each of the five use cases. Notably, we consider existing technologies that can be suitable candidates per use case because match the design requirements better.

### 3.1. Hospital Application

This use case will employ two different wireless protocols, namely Wi-Fi and XBee. The IEEE 802.11-based Wi-Fi protocol will primarily handle MiR100-to-camera communication. Wi-Fi infrastructure is already installed in some hospitals, and provides good coverage with relatively high data rates needed for transmission of live video streams. In addition, since both MiR100s are connected to the same network, non-safety related information and data will be transmitted between the MiR100s over the Wi-Fi connection. The Wi-Fi network can also be used for future communication between MiR100 and other hospital information systems of interest. For safety-critical MiR100-to-MiR100 communication, a small and inexpensive IEEE 802.15.4-based 2.4 GHz XBee-solution has been selected. This link will be used to safely coordinate and synchronize the movements of the two MiR100s. Although the over-the-air data rate of the XBee is limited to 256 kbps, this should be sufficient for this purpose.

The proposed setup of the autonomous hospital beds illustrated also in Figure 1 has been considered in some previous works that we discuss below. In [53], the authors provide an experimental testbed with mobile robots that can receive their position through a centralized camera. The images are processed in a central PC unit, which is able to send the position information to each robot over ZigBee standard. A distributed control algorithm based on event-triggered communications has been designed and implemented to bring the robots into the desired formation, where robots communicate to its neighbors only at event times. In [54], the authors provide a similar testbed setup that is used for localization and tracking using CMUcam3 modules mounted on static WSN nodes. A partially distributed approach was adopted and image segmentation was applied locally at each WSN camera node. The output of each WSN camera node, i.e., the location of the objects segmented on the image plane, is sent to a central WSN node for sensor fusion using an Extended Information Filter (EIF). To a similar direction, authors in [55] proposed a testbed setup, where the images captured synchronously by the cameras are processed at each node with the objective of extracting the essential information of the object. To cope with the usual low bandwidth of WSN, only this distilled information is sent through the WSN. The measures from all the cameras are integrated using information fusion methods such as maximum likelihood and extended Kalman filters. Finally, in [56], the authors also provide a cooperative control system of multiple robots using infrared cameras and image processing to facilitate the cooperative formation control. The above mentioned works are benchmarked in terms of distance among the mobile robots and their estimated location in a formation control. Due to the heterogeneity of the considered formation controls for each case, we can not compare their results. However, a general outcome is that localization errors could exist depending on demanding formation control and in case of traveling longer distances [57].

### 3.2. Maritime Application

This use case sees a bathymetry system based on a set of USV in addition to a manned vessel that drives and controls the measurement campaign. The terrestrial radio-systems, including very high frequency (VHF), high frequency (HF) and medium frequency (MF), are well established in the maritime community and are cornerstones of the mandatory global maritime distress and safety system (GMDSS) requirements for Safety of Life at Sea (SOLARS) vessels. Since 1970s, the mobile satellite communication has been used to the maritime community as well. Taking into consideration the cost and signal coverage, the bathymetry platoon uses the VHF radios as the primary communication channel. To ensure the reliability of communication, both the USV and manned vessel are also equipped with the transceiver for communication via the mobile network.

The Maritime Robotics (Maritime Robotics is a leading provider of innovative unmanned solutions for maritime operations in harsh environments: https://maritimerobotics.com/), i.e., autonomous boat platoons provider, has developed a VHF protocol named next generation ham radio (NGHam (https://github.com/skagmo/ngham)) and the corresponding radio system: “Owl VHF”.

NGHam specifies both physical (PHY) layer and media access control (MAC) layer functions [58]. The modulation schemes supported by NGHam are 2-GMSK (Gaussian Minimum Shift Keying) and 4-GMSK which result in different data rates of the channel, i.e., 9.6 Kbps and 19.2 Kbps, respectively. Although the available data rate of the VHF channel is low, it is sufficient when transmitting the critical information (e.g., vessel speed, position and course) in a regular periodic manner. However, the packet header does not include any field of packet type/priority. To distinguish the content of the packet, such as vessel voyage information or safety control command, some flag/type needs to be inserted into the payload field of the packet.

NGHam supports both carrier sense multiple access (CSMA) and time division multiple access (TDMA) schemes. Since message transmission between the USV and manned vessel requires the hard delay bound to ensure the safety of the cooperative bathymetry platoon, TDMA is a better option. It guarantees the worst-case end-to-end (E2E) delay through appropriately configuring the relevant parameters, such as TDMA frame length and slot size. TDMA requires synchronization among all users who access the shared channel to avoid interference caused by data transmission in consecutive slots. Synchronization can be realized referring to either some external clocks (e.g., global positioning system (e.g., GPS) or the internal clock of the master user.

### 3.3. Vehicular Applications

There are two potential solutions to support V2X communications: Dedicated Short Range Communication (DSRC)/Intelligent Transport Systems (ITS)-G5 and cellular network technologies such as 4G/5G. ITS-G5 generally refers to a wireless technology used for automotive and intelligent transportation system applications via short-range exchange of information among onboard units (OBUs) located inside the vehicles, RSUs placed on the side of the road, or handheld devices carried by pedestrians.

Cellular networks provide an off-the-shelf solution for this type of communications. 4G cellular networks is a scheduled network: transmission rates are granted by network scheduler, collisions are avoided and mutual interference is minimized. Quality-of-Service (QoS) can also be guaranteed (e.g., bit rate or delay) by allocating radio resources. On the other hand, some drawbacks that have been recognized (e.g., increased latency in case of high user density, non-optimized channel for small data, unavailability for out of coverage areas, etc.) are addressed by the Proximity Services (ProSe) feature, being specified within 3GPP [59]. ProSe, similarly to ITS-G5, allows user equipment to discover and communicate with each other directly within communication range, regardless of whether they are in or out of network coverage.The ProSe specifications do not cover the whole V2X requirements (it has been designed with the requirements of public safety and commercial consumer applications in mind). Enhancements are required for high speeds (e.g., in highway scenarios), guaranteed QoS and support for broadcast and unicast communications.

5G will most likely integrate into a heterogeneous network the already available communication technologies like LTE ProSe and IEEE 802.11p and will provide the necessary extensions to enable the future V2X use cases. 5G, as a general objective, will exploit safety, security and privacy support both from the infrastructure and application point of view. From the application point of view, the 5G integrated architecture will allow new business models characterized by services and applications ensemble with an increasing interaction, cooperation and complexity level as well as a great level of flexibility for service tailoring on customer demands. At the infrastructure level, the research aims to satisfy most vertical use case requirements, improving and enhancing the current technologies in an evolutionary scenario, thus solving the foreseen weakness of LTE and ProSe for the vehicular use case.

To support the requirements of different vehicular applications, each vehicle must be aware of the position, status and intention of its surrounding vehicles through message broadcasting. The European Telecommunications Standards Institute (ETSI) defines two types of messages: periodic Cooperative Awareness Messages (CAM) [60], and event-triggered Decentralized Environmental Notification Messages (DENM) [61]. CAMs include information such as geographical location, speed, and acceleration, and are only sent to a close neighborhood, as the validity of the information they contain is very limited in time. A large variety of C-ITS based safety applications are built upon the periodic exchange of CAMs, and their timely and reliable transmission is vital as a vehicle that continuously fails to deliver its beacon becomes invisible to its neighbors, which may result in potentially hazardous situations. Based on American standardization, CAMs are periodically generated, while ETSI recently decided upon a set of kinematic CAM triggering rules that trigger beacons when needed rather than keeping it strictly periodic. On the other hand, DENMs are only generated when an event of common interest occurs, and it is spread within an area of interest for the duration of the event.

An IEEE 802.11p network contains no access points or base stations, and consequently, will not experience coverage problems. This is the main benefit of IEEE 802.11p compared to other WLAN technologies. The supported ad hoc mode reduces delay, as messages do not have to take the detour around the access point or base station. ETSI is responsible for developing the whole protocol stack including vehicle-centric road traffic safety applications, whereas applications orienting towards road traffic efficiency utilizing road infrastructure are under the responsibility of CEN. ETSI has standardized a profile of IEEE 802.11p adapted to the 30 MHz frequency spectrum at the 5.9 GHz band allocated in Europe that today comprises one control channel and two service channels. Non-safety related applications are directed to a 20 MHz band at 5.855–5.875 GHz. The dedicated frequency bands have been divided into 10 MHz frequency channels. Due to the proximity of these bands to the frequency band used for ETC in Europe (5.795–5.805 GHz), ETSI TC ITS must also develop mitigation techniques to avoid to interfere with the ETC systems. There is no cost associated with using this frequency band (it is license free). However, EN 302 571 standard specifies requirements on output power limits, spectrum masks, etc.

A MAC protocol for a typical vehicular application has to be flexible enough to cope with high mobility and frequent topology changes. Therefore, the IEEE 802.11p MAC is based on a completely decentralized approach: the CSMA/CA random access MAC method used in IEEE 802.11 WLAN. The IEEE 802.11p MAC includes some enhanced features such as prioritized access to the channel by using queues with different arbitration interframe spaces (AIFS). This will ensure that data traffic with higher priority (e.g., video, IP telephony) has a higher probability of channel access compared to low priority traffic (e.g., background, best effort). However, the different QoS classes will not ensure timely channel access and thus, there will still be problems with collisions, especially during high utilization periods.

Regarding the intra-vehicle WSAN-(Wireless Sensor Actuator Network), the IEEE 802.15.4 first published in 2003 targeting low-rate WPANs, is perhaps the most paradigmatic technology supporting WSANs today. The protocol defines the physical and data-link layers and to complement it, several proposals such as the ZigBee, RPL, or 6loWPAN protocols were presented since its first release. More recently, to satisfy the requirements of emerging IoT applications, particularly in the industrial domain, the IEEE 802.15.4e amendment was proposed to complement the legacy IEEE 802.15.4-2011 standard. The IEEE 802.15.4e defines five MAC behaviors, instead of following a more conservative “one-size-fits-all” strategy. Hence, it improves its flexibility in accommodating different kinds of application requirements. In general, these new MAC behaviors are quite different from the ones considered in the legacy IEEE 802.15.4-2011. From the proposed MAC behaviors, the Deterministic Synchronous Multichannel Extension (DSME) is perhaps the closest to the legacy protocol, but nonetheless it brings significant enhancements to the IEEE 802.15.4 beacon-enabled mode by implementing multiple channel frequency hopping and Group Acknowledgments.

## 4. Safety and Security for SafeCOP Co-CPSs

SafeCOP use cases are representative of real application scenarios where safety and security play the key role. In the following, we analyze the safety and security requirements and solutions that are proposed for the five use cases of SafeCOP.

### 4.1. Autonomous Hospital Beds

An initial safety analysis of the concept has concluded that the network of external cameras is an enhancement to the basic functionality of the MiR100s, and should not be considered as part of the safety system. The MiR100-to-MiR100 communication, on the other hand, is an integral part of the safety system, and must be certified as safe according to the relevant safety communication standard. Unfortunately, there is currently no relevant safety standard for small, autonomous robots with wireless communication operating in a hospital environment. However, in the railway domain, there are similar challenges in signaling systems and train communication, where wires cannot be used due to the mobility of the application. The safe communication architecture will thus be based on the requirements of EN 50159 [62]. To avoid full safety certification of the communication protocol, it is proposed to use an end-to-end architecture with a safety layer inserted between the “black box” communication system and the safety application. In addition, since wireless communication by definition is an open communication system, which is vulnerable to attacks from actors with malicious intent, information security mechanisms (authentication, cryptography) are a prerequisite for achieving safe communication. The combination of threats random failures and external attacks will lead to one or more of the following basic message errors: repetition, deletion, insertion, resequence, corruption, delay and masquerade. In line with EN 50159, the XBee communication shall be enhanced with a safety layer implementing defenses against these threats. They consist of a protocol with the following parameters and functionalities: sequence number, time stamp, time-out, source and destination identifiers, feedback message, safety code, identification procedure, and cryptographic techniques.

### 4.2. Autonomous Boat Platoons

The primary scenario defined in this use case deals with one USV that is remotely controlled by a human operator located on another vessel. The wireless link connection between the USV and the manned vessel is maintained active by the continuous transmission of mode command messages from the manned vessel to the USV, to improve the reliability of the communication. If the message is not received within a given time-frame, the USV considers that the communication with the manned vessel is lost. Then, the USV will enter in fail-safe mode (e.g., stop). This mechanism is implemented at application level and independent of the underlying communication protocol.

A crucial safety requirement that the cooperative bathymetry platoon has to fulfill is to guarantee that a safe distance is maintained between the USV and the manned vessel. The acceptable distance between the USV and manned vessel is calculated as the maximum distance that allows messages carrying vessel voyage information be reliably and timely transmitted over the wireless network. Since the signal strength in maritime wireless networks is subject to perturbations due to the sea movement, safety messages are subject to packet loss at communication level. Therefore, sending messages in a periodic manner is applied to compensate packet loss. The vessels speed is typically slower than other transportation systems (e.g., cars, trains, etc.). Thus, missing one message is acceptable if the following one can be received correctly and timely.

Another safety requirement related to communication of safety-critical events emerges e.g., in the avoidance of potential collisions with obstacles such as another vessel, swimmer, or buoy. The manned vessel issues safety-control commands to the USV, which has to respond within a certain time to avoid collisions. To meet this requirement, the messages carrying control commands shall have higher priority than the messages sent out periodically. However, if a message carrying the safety-control command is lost during its transmission, the human operator may still have the chance to re-issue the same command if the message loss can be detected by timeout. It is worth noting that setting up a timeliness of the control command needs to take into account the movement of the USV and the distance to the obstacle. Thus, the timeliness of the control command may be varying from command to command.

Additional safety and security requirements have to be fulfilled to ensure safe USV operations [62,63]:

- *Messages authentication* ensures that the message is received in the same condition as it is sent out with no bits inserted, missing or modified. If the message is modified en route, then the receiver will certainly detect this. Without message authentication, the message, which is either corrupted or modified during transmission, carries the wrong information/command and may lead the USV to an unsafe-state, e.g., colliding with an obstacle.
- *Message timeliness* mechanism effectively limits the age of validly of delivered messages. Thus, if an attack diverts the validated messages for replay much later, the receiver can detect the delay introduced by this attack. Without timeliness constraint, the message which is not modified/corrupted but delayed may lead the USV to an unsafe-state. For example, if the command that the USV shall reduce the speed to avoid a potential collision is delivered to the USV too late, the USV may not have sufficient time to reduce its speed to avoid collision.
- *Message sequence* can be used to detect message loss, repetition and insertion. Without this mechanism, the attacker may intercept messages or insert malicious messages without being detected.

NGHam does not support any of the security mechanisms listed above. Therefore, the corresponding security functions need to be specified and implemented separately.

### 4.3. Vehicle Control Loss Warning

In the vehicle platooning scenarios, one vehicle may influence the behavior of other vehicles, and, since the consequences of a failure can harm human life, these systems are considered safety-critical and must be designed according to relevant methodologies to ensure safety, also from a communication perspective. The main goal in vehicle platooning is finding the best trade-off between performance (i.e., maximize speed and minimize vehicles reciprocal distance) and safety (i.e., avoid collision). The largest part of the literature focuses on advanced control schemes, without modeling the communication medium properly. Delay of communication is typically considered as a fixed delay or through probabilistic models. This allows the analytical derivation of string stability models [64] under some hypotheses of the dynamical system, but it may be unreliable under realistic conditions. Two branches are evident from the literature in this respect: the derivation of simple models of the delay bound that guarantees safety (see, e.g., section IV.C of [65]) and brute force simulation with visualization of safety regions under a reduced set of parameters [66,67]. Ongoing research addresses formal verification to extract evidence of safety conditions [68]. Authors in [69] apply machine learning for sensitivity analysis of safety conditions in platooning, under the constraint of no false negative, i.e., avoiding to predict safety (no collision) while collision happens in reality.

Regarding communications, and in particular safety, we identified several concerns. An unexpected interruption of the communication between two nodes can have an extremely negative impact in the safety of the platooning. A proper defense mechanism against erasure must be in place. Furthermore, communication must support response to violations on time (before a deadline), thus delivery time, must be ensured to bound the delay. Finally, these systems must also cope with RF interference without loss of main functionality. Although generic, this requirement aims at guaranteeing that the communication system is resilient to wireless interference on one hand (accidental or not), and can be robust to cope with an eventual attack, such as a DoS attack. We believe that, by relying on the EN 50159 black-channel approach, we will be able to address most of the safety issues for inter-vehicular communication, while escaping the need to certify the whole communication protocol, easing the use of COTS communication stacks. While this approach can mitigate several (if not all) of these safety constraints, there are still others requirements that must be addressed, particularly in what concerns QoS performance, to support the expected behavior of the platoon.

In a vehicular platoon, a lead vehicle that is responsible for managing the platoon’s moving directions and velocity, periodically disseminates control commands to following vehicles based on vehicle-to-vehicle communications. Inevitably, pushing vehicles to drive in close formation as the platoon requires low latency driving command transmission from the lead vehicle to the tail. Two critical challenges arise in the inter-vehicle wireless communication. The first challenge is that signal fading induces dynamic wireless channels, which causes command loss at the receiver. This command loss is especially crucial in vehicular platoons since command reception at each vehicle highly depends on the reception of its preceding vehicle. Moreover, command loss at preceding vehicles can impact the command dissemination due to retransmissions, which may lead to accidents due to lack of timely updates. The second challenge is the possibility of assigning the exact transmit rate to each vehicle in the platoon. Although a high transmit rate achieves low transmission latency for each vehicle, increasing the transmit rate results in increasing the receiver’s bit error rate (BER) at a given Signal-to-Noise Ratio (SNR). Accordingly, the vehicle with high BER spends longer time on command retransmissions, which prolongs dissemination latency of the platoons. Therefore, allocating the transmit rate without a proper adaptivity leads to command dissemination latency performance degradation. In [70], we proposed a low-latency driving command dissemination (LCD) algorithm to adapt the transmit rate (i.e., modulation) allocation of vehicles as such that the latency of command dissemination in the platoon is minimized under guaranteed BER. We proved that the LCD algorithm achieves computation time complexity of O(NM2), where N and M are the number of vehicles and modulation levels, respectively. The simulation results show that LCD significantly improves the dissemination rate by 50.9% existing algorithms. Moreover, LCD also approximates the lower bound of dissemination latency with the maximum gap of up to 0.2 s.

Regarding security, each node’s wireless communications must also be encrypted. Data exchange among nodes must be properly secured to prevent unauthorized access and alteration of the message content for malicious purposes. Due to broadcast nature of radio channels, disseminating sensory data is vulnerable to eavesdropping, and message modification from an illegitimate eavesdropper. To improve communication security in CPS, using a shared secret key for data encryption/decryption is crucial to support data confidentiality, integrity, and sender authentication. Key generation based on the randomness in a wireless fading channel is a promising approach [71], where two sensor nodes extract secret bits from the inherently random spatial and temporal variations of the reciprocal wireless channel between them. However, while previous works on fading channel based secret key generation mainly focused on improving the secret bit generation rate between a pair of sensor nodes (by exploiting temporal and spatial variations of radio channel, multiple antenna diversity, or multiple frequencies), the problem of unanimity of the generated key for the real-time data dissemination remained a challenge. To address this, we presented in [72] a new data dissemination security protocol that quantizes the estimated received signal strength (RSS) measurements. The quantization intervals are cooperatively adapted to reduce secret bit mismatch rate (BMR). The secret key generated by our protocol is based on channel randomness over multiple hops, the eavesdropper at a different location experiences independent channel fading, which is not able to obtain the same key. In addition, the proposed protocol can be applied to more critical systems, as the secret key is generated in a distributed manner, eliminating a single point of failure.

In addition to inter-vehicular communications, its wireless intra-vehicular counterpart is also being addressed. In-vehicle wireless networks have been recently proposed with the goal of reducing manufacturing and maintenance cost of a large amount of wiring harnesses within vehicles [73,74]. The wiring harnesses used for the transmission of data and power delivery within current vehicles may have up to 4000 parts, weigh as much as 40 kg and contain up to 4 km of wiring. Eliminating these wires would additionally have the potential to improve fuel efficiency, greenhouse gas emission, and spur innovation by providing an open architecture to accommodate new systems and applications. Interestingly, in [75], Volvo group trucks technology presented a practical design of an in-vehicle WSN, using the IEEE 802.15.4 TSCH protocol as the MAC protocol. This work uses a network with only 10 nodes, while vehicles have the potential to use a much higher number of wireless sensors. DSME has better performance as the number of nodes increases and is probably the most flexible MAC behaviour from all the IEEE 802.15.4e proposals. Its multi-superframe structure allows for the transmission of both periodic as well sporadic traffic, while still supporting a fast reconfiguration of the DSME-GTS schedule. Several work has already been done regarding this protocol regarding the evaluation of its performance limits [76], and behavior [9], and some performance improvements are on the way [77].

### 4.4. Vehicles and Roadside Unit (RSU) Interaction

In this use case, safety and security risks are related to creating links and communication between different actors: service providers, vehicles and roadside units. Roadside unit is hosting the road weather station measurements and sensors, vehicle unit the embedded vehicular sensors and service provider the general service data. In each of the elements, the same security risks remain: identification of counterpart, validating the runtime operation of sensors and services and avoiding malfunctions due to interference with parallel communication. Other safety aspects are related to ensure the complete data exchange procedures in local area vehicular networking, when vehicles are passing each other or roadside unit. IEEE 802.11p operates in dedicated 5.875–5.905 GHz band in Europe. In 3G, the operator is hosting the communication parameters in each link, ensuring the quality of service. Therefore, the communication failures are typically caused by capacity overload. Communication can be disturbed by intentional interference as well, which must be taken into account when aiming to ensure safety and security.

### 4.5. V2I Cooperation for Traffic Management

Fundamental safety requirements for this use case are as follows:

- *Early detection of communication errors*, i.e., packet loss, packet insertion, packet replication, packet inversion.
- *Bounding to a known upper limit the WSN communication latency to the roadside unit*.
- *Executing image processing for VCA on two different HW components* (i.e., locally on the camera and remotely on the Network Video Recorder).

From the safety standpoint, a thorough hazard and risk analysis has been conducted, employing both ISO 26262 and STAMP [31] methodologies. The former identifies 35 hazard conditions, related to incorrect behaviours from a given subsystem. Since in ISO 26262 all components are considered in isolation, a safe state needs to be defined every time a component cannot guarantee an Automotive Safety Integrity Level at the “Quality Management” rank (i.e., the most safe). For the sake of brevity, we exemplify only the most dangerous condition identified, that of the on-board unit of the vehicle performing a braking action when no danger is actually present. Since this action could actually lead to accidents (ASIL-C), the runtime manager needs to inhibit the transmission of commands from the on-board unit to the control CAN bus. The latter identified 11 system-level risks. While it may appear that ISO 26262 provides a more detailed analysis of risks, it must be taken into account the fact that STAMP risks are system-level by nature, and are therefore relevant when considering the combined effects of multiple subsystems. As an example, the STAMP analysis highlighted as a key hazard the violation of integrity level of the V2I system, and led to the need to include appropriate detection tools (e.g., those developed in [78] at system level to preserve the operational status from external intrusion.

In terms of road safety, the most critical function provided in this use case is the image processing performed at the RSU-C and forwarded at the VCA platform, in order to detect dangerous situations and accidents in the monitored area and alert the LCU/RSU. This unit will apply then a specific countermeasure (GLOSA, ATL-S) over the relevant traffic zone. For this application, the requirements of latency and reliability of the wireless communication channel are of utmost importance, since the warning messages and countermeasures at the traffic lights should reach the destination at the due time. A full analysis of the computational workload is reported in [52]. It is worth noting, however, that such analysis is typically dependent on the specific road intersection scenario, and the resources available for the analysis must therefore be tuned to minimize the risk of exceeding the required latency.

From the security standpoint, authentication and encryption shall be applied to all communications; any non-authenticated and/or non-encrypted transmission cannot be automatically considered trustworthy. A non-authenticated communication may come to the control center from either a traffic light, a car or a WSN node. In the former case, the breach of authenticity is a critical issue, and the Co-CPS should switch to a safe mode. In the latter case, the non-authenticated information may simply be discarded, or be used in the decision process only if it is corroborated by coherent information coming from authenticated entities. Similar considerations apply for breaches of integrity. Regarding breaches of availability, the safety of the system clearly depends on the timeliness of the information. If breaches of availability are detected, the runtime manager should aptly react by moving to safer states. Therefore, we identify three service levels: (1) integrity, authenticity and availability are ensured for any message; (2) integrity, authenticity and availability are ensured for messages exchanged among infrastructure components only; (3) at least one infrastructure component cannot ensure integrity, authenticity and availability. ATL-S and GLOSA can be applied at service levels 1 and 2, but no cooperative activity can take place at level 3. The runtime manager will therefore inhibit transmissions from the control unit when level 3 is reached, returning the system to the safe state (i.e., the non-cooperative system). At level 1, functions beyond the current goals of the V2I scenario (e.g., platooning) could be supported as well.

In the V2I scenario, critical aspects of security are primarily related to ensuring that communications where one endpoint has limited computation capabilities, and is possibly exposed to attackers, are suitably protected while remaining within the resource and time budget. To this end, the WSN security mechanisms used for V2I cooperation in this use case employ a hybrid cryptography scheme (TAKS) derived as extensions of the contributions in [7,8,79]. Furthermore, the trade-off between security and latency can be tuned by employing tools such as [80], which allow for selecting appropriate countermeasures to side-channel attacks.

## 5. 5G Open Challenges

In this section, the open challenges raised from the architectures of the SafeCOP use cases presented above are discussed in relation to 5G use cases. The reason to choose 5G to discuss about the technical challenges is the fact that 5G system can accommodate (from wireless communications standpoint) most of the SafeCOP use cases as identified from above discussion. Notably, 5G promises to facilitate new vertical industries through network slicing and network softwarization [81]. Our study below proves that 5G communications can facilitate most of the innovative CPS related use cases of SafeCop project. Safecop had as a major objective to provide cooperative safe CPS using wireless communications technologies and 5G could be considered the most promising candidate solution.

Work on 5G is currently in progress to meet the 2020 objectives of a ready-to-use technology. The European Union promoted the METIS project in 2012 [82,83], followed by the 5GPPP program [84], as well as other significant projects like e.g., CROWD [85]. The NGMN Alliance, which was founded by major mobile operators in 2006, provided an important contribution to 5G by publishing the NGMN 5G White Paper in March 2015 [86].

As the future cellular network’s evolution, the 5G ecosystem is a multi-provider/multi-tenant environment. It is designed as a heterogeneous network supporting business and applications services [87]. It is structured in a set of layers divided into two basic groups:

1. *The higher service-centric layers* modeling the business implementations driven by the vertical use cases.
2. *The lower network-centric layers* representing the physical implementation and its abstraction based on software network technologies like software defined networking (SDN) and Network Function Virtualization (NFV).

The 5G protocol stack is based on IPv6, as defined in [88,89,90]. Network flexibility is obtained by the establishment of slices, configured for the purposes of specific application scenarios. Slicing allows the creation of multiple virtual networks on top of a shared physical infrastructure. They allow specific operators to offer ITS related services, ensuring the right prioritisation of communication channels (e.g., road monitoring for safety over other INTERNET traffic). Additional issues are network coverage and densification, where the latter consists in adding more cell sites to increase the available network capacity. Device-to-Device communications and the concept of virtual cells contribute to addressing both issues.

As 5G is designed according to specialized application requirements, its applicability to the SafeCOP use cases should be accurately discussed.

For the maritime use case, the radio high frequency transmission could suffer from hard degradation and interferences due to water surface and possible adverse weather conditions, challenging the use of 5G technology. Satellite communication is still possible, but latency and response time are generally not acceptable except for critical situations as a backup solution.

In case of a healthcare use case, the currently foreseen protocols like 802.15.4 and 802.11 are very suitable for indoor and intra-robot communications. 5G can be considered exploiting the “femtocell” indoor solution and integrating the system to a wider infrastructure for possible future expansion or effective service distribution.

The set of automotive use cases will take a great advantage by 5G evolution, since the research and improvement area of 5G aim to solve the limitations of current mobile and Ethernet-based technologies (i.e., LTE Proximity Service and 802.11p) [61,91,92,93,94].

Table 1 summarizes the 4G and 5G technology challenges related to SafeCOP use cases. We then outline how 5G standards may meet SafeCOP requirements and the open challenges ahead.

### 5.1. Autonomous Hospital Beds

With the idea of connecting different types of clinical devices to its network, 5G technology provides a pervasive environment in hospitals well beyond the one envisaged by this use case, specifically related to the automated management of hospital beds. In the health care sector, robots can be used to transport specimens, drugs, and bedlinen to wards, labs, pharmacies, and depositories, offloading repetitive low level tasks from skilled hospital staff as envisioned in [95]. Such a use case can be realized through the mission critical X (MCX) 3GPP services, like data and video introduced in [96,97]. The MCX 3GPP services apply to both aerial or terrestrial unmanned vehicles, including drones and robots. The Autonomous Hospital Beds use case focuses on robots that can be assimilated to terrestrial unmanned vehicles embedding 5G chipsets with MCX (MCData/MCVideo) air interface enabled. More ideas about robotics applications in healthcare within the 5G ecosystem can be found in [98], where robots are considered stand-alone configuration instead of a non-standalone new radio (i.e., 5G) application. To enable hospital bed use case in [96], the MCX 3GPP specifications integrates the following requirements:

1. MCData Service shall enable the control of robots;
2. MCData Service shall provide a common transmission framework to use and control robots;
3. MCData Service shall provide a default control latency depending on the robots type under (400 ms for a terrestrial unmanned vehicle).

The split between network latency and robot latency is left open for future research. Similar requirements can be found in [97] for video transmission, where MCVideo is coordinated with the MCData to give the most suitable priority to the control of the robots. It is evident that such design requirements can be used also to hospital beds, which can be equipped with both data/video transmission capabilities by implementing the MCX 5G interface. Since the 5G MCX interface is out of the scope of this paper, we refer the interested reader to the 3GPP web page in [99].

### 5.2. Autonomous Boat Platoons

Finding the right position for this use case in 3GPP standardization is not straightforward. In principle, we can make the assumption that autonomous shipping are enabled by a joint initiative of satellite operators and space agencies, where 5G satellite-related technology drive the autonomous shipping vision [100]. Enabling network availability to moving platforms such as passenger vehicles, aircraft ships, trains, buses, etc., is a requirement for the new radio interface for non-terrestrial networks (NTN) [101]. In [102], the authors discuss a sort of ship-mega constellation, which is similar to boat platooning, based on multi-hop communications enabled by both 5G and satellite communications. A clear vision towards autonomous shipping is presented in [100], based on satellite communications, linked to the joint industry project called Advanced Autonomous Waterborne Applications (AAWA) led by Rolls Royce. Unfortunately, 5G is not included.

### 5.3. Vehicular Applications

#### 5.3.1. Vehicle Control Loss Warning

In [103], the control loss warning is considered as a use case, where it enables a host vehicle to broadcast a self-generated control loss event to surrounding Remote Vehicles (RV). Upon receiving such event information, an RV determines the relevance of the event and provides a warning to the driver.

#### 5.3.2. Vehicles and Roadside Unit (RSU) Interaction

A few V2X use cases towards 5G were initially introduced in [103,104] followed with details about enhanced V2X 5G services. In particular, requirements for use cases that rely on vehicles and roadside unit interaction such as platooning and advanced driving is provided in [104]. The following summary of requirements related to vehicles-RSU interaction is included:

- Between User Equipment (UE) supporting V2X application and RSU via another UE supporting V2X application with conditionally and fully automated driving, where the payload message could vary from 50–1200 bytes and latency to 20–50 ms.
- Between RSU and UE supporting V2X application with again partially or fully automated driving, where the payload message is lower around 100 bytes and latency to 10–50 ms.

A technology that could be useful for future 5G for most of the V2X services is network slicing [105]. The RSU can be also employed in Mobile Edge Computing (MEC), as a host of e.g., MEC server [106]. However, details are lacking in case of vehicles to RSU interaction and only high-level ideas are proposed towards the use cases reported in [104].

#### 5.3.3. V2I Cooperation for Traffic Management

A mixed-use traffic management use case is proposed in [103], which includes various transportation modes (e.g., automobile, train, bicycle, and pedestrian), several traffic densities, and different environmental conditions. It is shown that a V2X system would need the flexibility to adapt to changing attributes such as vehicular traffic density, rates of speed, angles of approach, and weather conditions which all may impact the optimal range and transmission rate in a specific situation. However, the description of such a mixed-use case is not sufficiently detailed in [103,104]. Looking into the related research works, we have identified one recent contribution on traffic management [107]. It deals with urban traffic management to mitigate traffic congestion. A new architecture is defined, based on different layers, such as: environmental sensing, communication, mobile edge computing and remote core cloud server. 5G SDN and mobile edge computing are considered key enabling technologies. SDN offers high-bandwidth communication service with flexibility and programmability, thus providing agility to sensing operations. Other technologies are also listed for future research such as vehicle localization, data pre-fetching strategy, traffic prediction and traffic lights control.

## 6. Conclusions

This survey provides an overview of the technology requirements for future Co-CPS in terms of wireless communication and safety as analyzed within the framework of SafeCOP European project. We focused on highlighting the main wireless communication technologies currently available and evaluated their compliance to the safety requirements of Co-CPSs in terms of dependability, security and timeliness. The survey states the general recommendations deciding about the use of wireless communication technologies in Co-CPSs and illustrates them through real-world use cases. Furthermore, the suitability of 5G technology to the SafeCOP use cases has been evaluated and discussed. Although its applicability is not straightforward, 5G technology offers clear benefits in several aspects but is challenging for some others. Through the presented analysis, the SafeCOP project is developing a reliable approach to safety assurance in cooperating cyber-physical systems. This approach is expected to represent a reference architecture able to fulfill the safety and security requirements proper for Co-CPSs as they have been individuated by the thorough analysis presented in this article.

## Figures and Tables

**Table 1:** 4G and 5G technology challenges in SafeCOP.

Use Case	4G	5G
Heathcare	Energy efficiency issue on radio interfaces	Femtocell solution for indoor comms Support scalability
Maritime	Radio signal degradation	Satellite transmission but latency concerns
Vehicle control loss warning	ProSe to be upgraded	natively supported
Vehicles and roadside units interaction	ProSe to be upgraded	natively supported
V2I cooperation for traffic management	latency concerns	natively supported
